# Modulation of transient receptor potential vanilloid 4-mediated membrane currents and synaptic transmission by protein kinase C

**DOI:** 10.1186/1744-8069-5-5

**Published:** 2009-02-10

**Authors:** De-Shou Cao, Shuang-Quan Yu, Louis S Premkumar

**Affiliations:** 1 Department of Pharmacology, Southern Illinois University School of Medicine, Springfield, IL 62702, USA

## Abstract

**Background:**

Transient receptor potential Vanilloid (TRPV) receptors are involved in nociception and are expressed predominantly in sensory neurons. TRPV1, a non-selective cation channel has been extensively studied and is responsible for inflammatory thermal hypersensitivity. In this study, the expression and function of TRPV4 have been characterized and compared with those of TRPV1.

**Results:**

Immunohistochemical studies revealed that both TRPV1 and TRPV4 were co-expressed in dorsal root ganglion (DRG) neuronal cell bodies and in the central terminals of laminae I and II of the spinal dorsal horn (DH). In Ca^2+ ^fluorescence imaging and whole-cell patch-clamp experiments, TRPV1- and TRPV4-mediated responses were observed in a population of the same DRG neurons. Sensitization of TRPV1 has been shown to be involved in inflammatory pain conditions. Incubation with phorbol 12, 13-dibutyrate (PDBu), a PKC activator, resulted in a significant potentiation of TRPV4 currents in DRG neurons. In TRPV4 expressing HEK 293T cells, PDBu increased 4α-phorbol 12, 13-didecanoate (4α-PDD)-induced single-channel activity in cell-attached patches, which was abrogated by bisindolylmaleimide (BIM), a selective PKC inhibitor. TRPV4 is also expressed at the central terminals of sensory neurons. Activation of TRPV4 by 4α-PDD increased the frequency of miniature excitatory post synaptic currents (mEPSCs) in DRG-DH neuronal co-cultures. 4α-PDD-induced increase in the frequency of mEPSCs was further enhanced by PDBu. The expression of TRP channels has been shown in other areas of the CNS; application of 4α-PDD significantly increased the mEPSC frequency in cultured hippocampal neurons, which was further potentiated by PDBu, whereas, TRPV1 agonist capsaicin did not modulate synaptic transmission.

**Conclusion:**

These results indicate that TRPV4 and TRPV1 are co-expressed in certain DRG neurons and TRPV4 can be sensitized by PKC not only in DRG neuronal cell bodies, but also in the central sensory and non-sensory nerve terminals. Co-expression of TRPV1 and TRPV4 ion channels, their modulation of synaptic transmission and their sensitization by PKC may synergistically play a role in nociception.

## Background

Transient receptor potential (TRP) channels are involved in initiating and transmitting sensory information from the periphery to the CNS. TRPV4 is a Ca^2+ ^permeable non-selective cation channel, first described as an osmosensor [1] and recently has been shown to be activated by heat (> 27°C), low pH, phorbol ester derivative 4α-phorbol 12, 13-didecanoate (4α-PDD), endocannabinoids and arachidonic acid (AA) metabolites [2-6]. It is expressed in multiple tissues, including lung, kidney, heart, gut, sensory neurons, sympathetic neurons, vascular smooth muscle cells and endothelial cells [1,2,7-9]. The higher expression levels of TRPV4 in keratinocytes indicate that contribution of TRPV4 to thermal sensation is not restricted to sensory neurons [10]. TRPV4 null mice displayed impaired osmotic regulation, suggesting that TRPV4 is necessary for maintaining osmotic equilibrium in mammals [11]. It has been reported that inflammatory and thermal hyperalgesia induced by carrageenan is attenuated in TRPV4 knockout mice [12]. TRPV4 has been shown to be required for hypotonicity-induced nociception and chemotherapy-induced neuropathic pain [13,14]. Furthermore, in models of painful peripheral neuropathy induced by vincristine chemotherapy, alcoholism and diabetes, mechanical hyperalgesia was attenuated by intrathecal injection of TRPV4 antisense oligodeoxynucleotides, and the similar effect was also observed in TRPV4 knockout mice [15]. TRPV4 deficient mice exhibited impaired acid- and pressure-induced nociception [5]. TRPV4 has been shown to contribute to visceral hypersensitivity [16,17]. These studies suggest that TRPV4 is involved in both inflammatory and neuropathic pain and play a key role in mechanical nociception.

Vascular endothelial cells, renal collecting duct cells and vascular smooth muscle cells expressing TRPV4 are particularly susceptible to cell swelling-induced Ca^2+ ^influx that can be blocked by ruthenium red, a nonspecific blocker of TRP channels [4,7,18,19]. Cell swelling also activates phospholipase A2 (PLA2) and produces AA. AA and its cytochrome P450 metabolite 5',6'-epoxyeicosatrienoic acids (EETs) activate TRPV4 [6]. Further evidence for this pathway is shown by the ability of PLA2 blockers to inhibit hypotonicity-induced Ca^2+ ^influx and membrane current [20]. In behavioral studies, hypotonicity-induced nociception has been shown to involve PKA- and/or PKC-mediated phosphorylation [21]. Modulation of TRPV1 by PKC has been extensively studied; in this study, we will address the modulation of 4α-PDD-induced TRPV4 function by PKC.

Activation of TRPV1 modulates synaptic transmission at the first sensory synapse between DRG and DH neurons [22-25]. TRPV1 has also been reported to modulate synaptic transmission in certain regions of the brain [26-29]. The activation of TRPV4 facilitated substance P (SP) and calcitonin gene related peptide (CGRP) release from the central terminals of primary neurons in the spinal cord [30]. Although it has been demonstrated that TRPV4 is expressed in sensory and non-sensory neurons, the role of TRPV4 in the modulation of synaptic transmission remains to be studied.

In this study, we show that TRPV4 is co-expressed with TRPV1 in DRG and dorsal horn laminae I and II. We have also found that TRPV4-mediated channel activity induced by 4α-PDD is further augmented by activation of PKC. In addition, TRPV4 activation modulates synaptic transmission in DRG-DH co-cultures and hippocampal neuronal cultures, which is further enhanced by the activation of PKC.

## Methods

### Immunohistochemistry

Five weeks old Sprague-Dawley rats were anesthetized by isoflurane and perfused with 4% paraformaldehyde. Samples of lumbar segments of the spinal cord and DRG were harvested and quickly frozen. The spinal cord and DRG were cut into 20 and 10 μm sections, respectively. The sections were incubated with polyclonal goat anti-TRPV1 antibody (Calbiochem, PC 420, 1:100) and rabbit anti-TRPV4 antibody (Alomone, Israel, 1:100) for 2 hrs at room temperature, then incubated with Rhodamine Red ™-X donkey anti-goat IgG (Jackson, 711-295-152, 1: 100) and FITC donkey anti-rabbit IgG (Jackson, 715-095-151, 1: 100) for 1 hr at room temperature. Images were taken by a confocal microscope.

### DRG, DRG-DH and hippocampal neuronal cultures

DRG, DH and hippocampal neurons were isolated from embryonic day 18 (E18) rat embryos, triturated and cultured in Neurobasal medium (Invitrogen, Carlsbad, CA). The adult DRG neurons were dissociated from 5 weeks old rats. The serum-free medium was supplemented with B-27 and glutamine (Gibco Invitrogen, Grand Island, NY) and the neurons were plated on poly-D-lysine-coated glass coverslips. The neurons were used after 2 weeks growth in culture.

### HEK 293T cell culture and transfection

Human embryonic kidney 293T cells were cultured in DMEM with 10% fetal bovine serum and penicillin (50 units/ml)-streptomycin (25 μg/ml) (Gibco Invitrogen, Grand Island, NY). TRPV4 cDNA and GFP cDNA were co-transfected into HEK 293T cells with Lipofectamine 2000 reagent following manufacture's protocol (Invitrogen, Carlsbad, CA). The fluorescent cells were used for recording currents 24 hrs after transfection. The non-fluorescent cells were used as a negative control.

### Ca^2+ ^fluorescence imaging

Adult dissociated rat DRG neurons plated on glass coverslips were incubated with 3 μM Fluo-4 AM (Invitrogen, Eugene, OR) for 30 min at 37°C and washed with physiological buffer containing the following (in mM): 140 NaCl, 5 HEPES, 2 CaCl_2_, 1 MgCl_2_, 2.5 KCl, 2 Lidocaine, pH 7.35. Fluo-4 was excited at 488 nm, and emitted fluorescence was filtered with a 535 ± 25 nm bandpass filter. The ratio of the fluorescence change *F*/*F*_o _was plotted to represent the change in intracellular Ca^2+ ^levels.

### Whole-cell current recording

DRG neurons grown on poly-D-lysine-coated coverslips were used for recording TRPV1 and TRPV4 currents. For whole-cell patch-clamp recordings, the bath solution contained (in mM): 140 Na gluconate, 5 KCl, 10 HEPES, 1 MgCl_2_, 1.5 EGTA, pH adjusted to 7.35 with NaOH and the pipette solution contained (in mM): 140 K gluconate, 5 KCl, 10 HEPES, 2 MgCl_2_, 10 EGTA, 2 K_2_ATP, 0.5 GTP, pH adjusted to 7.35 with KOH. Currents were recorded using an Axopatch 200B integrating patch-clamp amplifier (Axon Instruments Inc., Union City, CA). Data were digitized (VR-10B; InstruTech, Great Neck, NY) and stored on videotape. For analysis, data were filtered at 2.5 kHz (-3 dB frequency with an eight-pole low-pass Bessel filter; LPF-8; Warner Instruments, Hamden, CT) and digitized at 5 kHz. Current amplitudes were measured using Channel 2 (software kindly provided by Michael Smith, Australian National University, Canberra, Australia).

### Single-channel current recording

The cell-attached patch-clamp technique was used to record single-channel currents. The bath solution contained (mM): K gluconate 140, KCl 2.5, MgCl_2 _1, HEPES 5, EGTA 1.5, pH adjusted with KOH to 7.3. The patch pipettes were made from glass capillaries (Drummond, Microcaps), coated with Sylgard (Dow Corning, Midland, MI, USA). The patch pipettes were filled with a solution that contained (mM): Na gluconate 140, MgCl_2 _2, HEPES 10, EGTA 1, pH adjusted with NaOH to 7.35. Currents were recorded using an Axopatch 200B integrating patch-clamp amplifier (Axon Instruments Inc., Union City, CA). Data were digitized (VR-10B; InstruTech, Great Neck, NY) and stored on videotape. For analysis, data were filtered at 10 kHz (-3 dB frequency with an eight-pole low-pass Bessel filter; LPF-8; Warner Instruments, Hamden, CT) and digitized at 50 kHz. The data were analyzed using Channel 2 and QUB (State University of New York at Buffalo, NY)

### Synaptic current recording

DRG, DH and hippocampal pyramidal neurons were identified by their morphology. The extracellular bath solution contained (in mM): 130 NaCl, 5 KCl, 1 MgCl_2_, 1 CaCl_2_, 10 HEPES, 5 D-glucose, pH 7.35 and the pipette solution contained (in mM): 140 K gluconate, 2 MgCl_2_, 1 EGTA, 10 HEPES, 1 ATP, pH adjusted to 7.35 with KOH. In order to record fast excitatory postsynaptic currents, neurons were voltage-clamped (Axopatch 200B, Axon Instruments Inc., Union City, CA) at -60 mV (close to E_Cl_). While recording mEPSCs the bath solution contained lidocaine (10 mM). The data were filtered at 2.5 kHz and digitized at 5 kHz. Data were analyzed using Mini Analysis Program (Synaptosoft, Decatur, GA). The amplitude and frequency of the events were determined from 30 s data segments.

### Chemicals and reagents

B27, glutamine, fetal bovine serum and penicillin-streptomycin were obtained from Gibco Invitrogen, Grand Island, NY. Fluo-4 AM was obtained from Invitrogen, Eugene, OR. Lipofectamine 2000 reagent was obtained from Invitrogen, Carlsbad, CA. All other chemicals used in this study were obtained from Sigma (St. Louis, MO).

### Data analysis

Data are represented as mean ± S. E. M. (Standard Error of the Mean) Kolmogorov-Smirnov (KS) test was used to compare the cumulative probability for inter-events and amplitude between different groups. Student's *t*-test and one way analysis of variance (ANOVA) were used for statistical comparisons and the significance is considered at *P *< 0.05.

## Results

### Expression and co-expression of TRPV4 in DRG neurons

**Figure 1:**
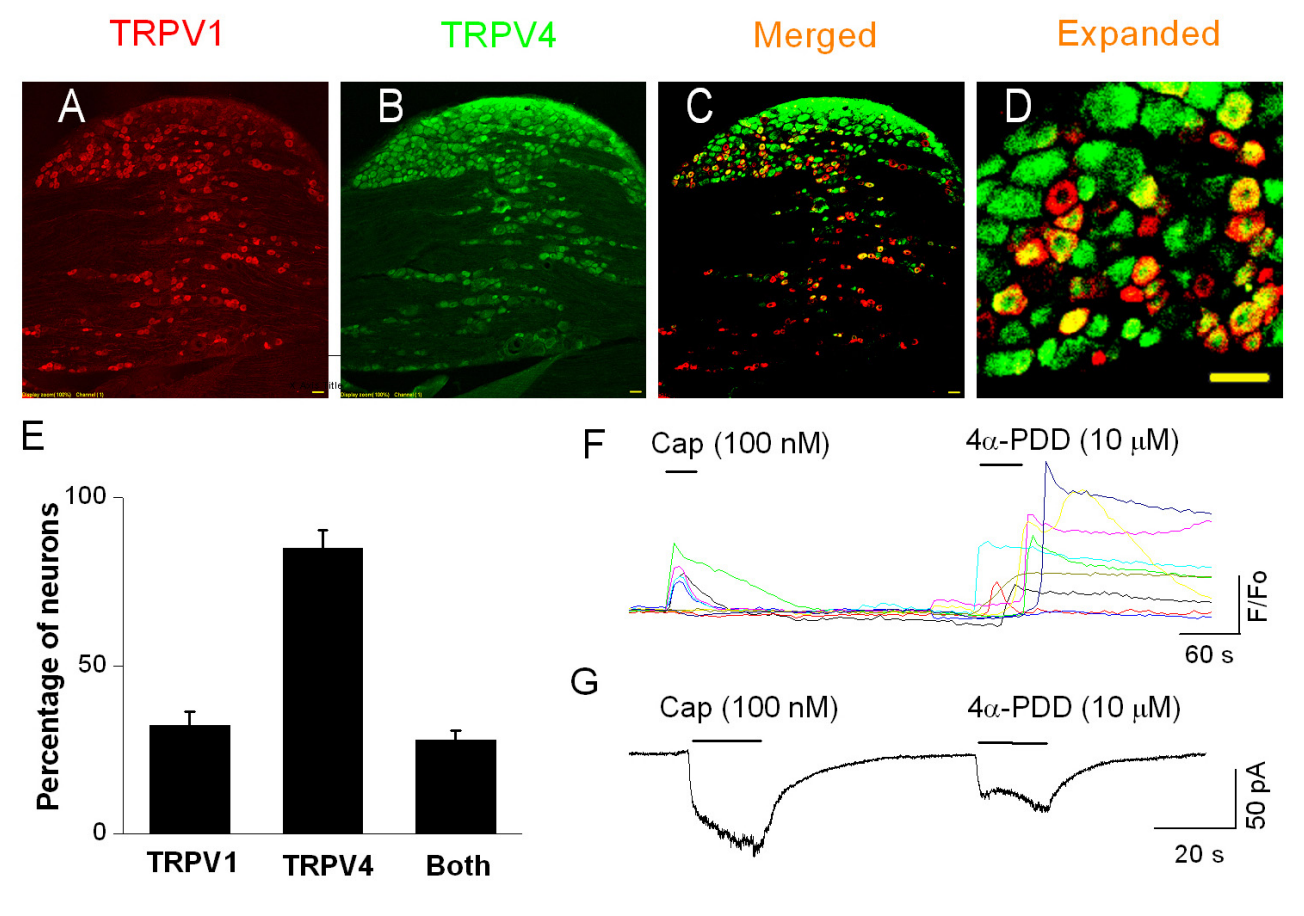
**TRPV1 and TRPV4 are co-localized in dorsal root ganglion (DRG) neurons**. **A**. Immunostaining of TRPV1 in DRG. **B**. Immunostaining of TRPV4 in DRG. **C**. Merged images of A and B. **D**. Expanded merged image. Bar = 50 μm. **E**. Summary graph showing the expression of TRPV1, TRPV4 and the extent of co-expression (*n *= 4). **F**. Ca^2+ ^fluorescence imaging showing capsaicin- and 4α-PDD-induced responses in the same neurons. **G**. Whole-cell current trace showing capsaicin- and 4α-PDD-mediated current responses in the same neuron.

Using immunohistochemical, patch-clamp and Ca^2+ ^fluorescence imaging techniques, we studied the expression and co-expression of TRPV4. Double immunostaining of sections from whole DRG revealed that 33.7 ± 2.4% of neurons expressed TRPV1, 88.5 ± 4.8% of the neurons expressed TRPV4, and 27.9 ± 2.8% of the neurons expressed both TPRV4 and TRPV1 (*n *= 4 rats, at least 3 slices from each animal) (Fig. 1A, B, C, D, E). The specificity of antibodies has been confirmed by a single band in Western blots from samples of HEK 293T cells heterologously expressing TRPV1 or TRPV4 (data not shown). Similarly, in Ca^2+ ^fluorescence imaging from adult dissociated neurons, 37.6 ± 3.9% of neurons responded to capsaicin, 64.2 ± 5.4% of neurons responded to 4α-PDD and 29.5 ± 3.2% of neurons responded to both, which is consistent with the immunostaining data (Fig. 1F). Mostly, the co-expression of TRPV1 and TRPV4 was observed in small and medium diameter neurons.

Then, we used whole-cell current recordings and determined whether TRPV1- and TRPV4-mediated currents can be elicited in the same neuron. In embryonic rat DRG neuronal cultures, 51/73 neurons responded to both capsaicin (100 nM) and 4α-PDD (10 μM) (Fig. 1G). These results indicate that TRPV1 and TRPV4 are co-expressed in DRG neurons. A higher number of neurons co-expressing TRPV1 and TRPV4 in whole-cell current recording as compared to immunostaining or Ca^2+ ^fluorescence imaging techniques may be due to the bias of selecting small diameter neurons or differential expression of these two channels between embryonic and adult DRG neurons.

### Modulation of TRPV4 by PKC

Several TRP channels have been shown to be modulated by PKC, which contributes to inflammatory pain [31]. There are suggestions that TRPV4 may be modulated/activated by PKC [32,33]. In order to determine the modulation of 4α-PDD-induced TRPV4 currents by PKC, we used patch-clamp technique and recorded whole-cell and single-channel currents.

**Figure 2:**
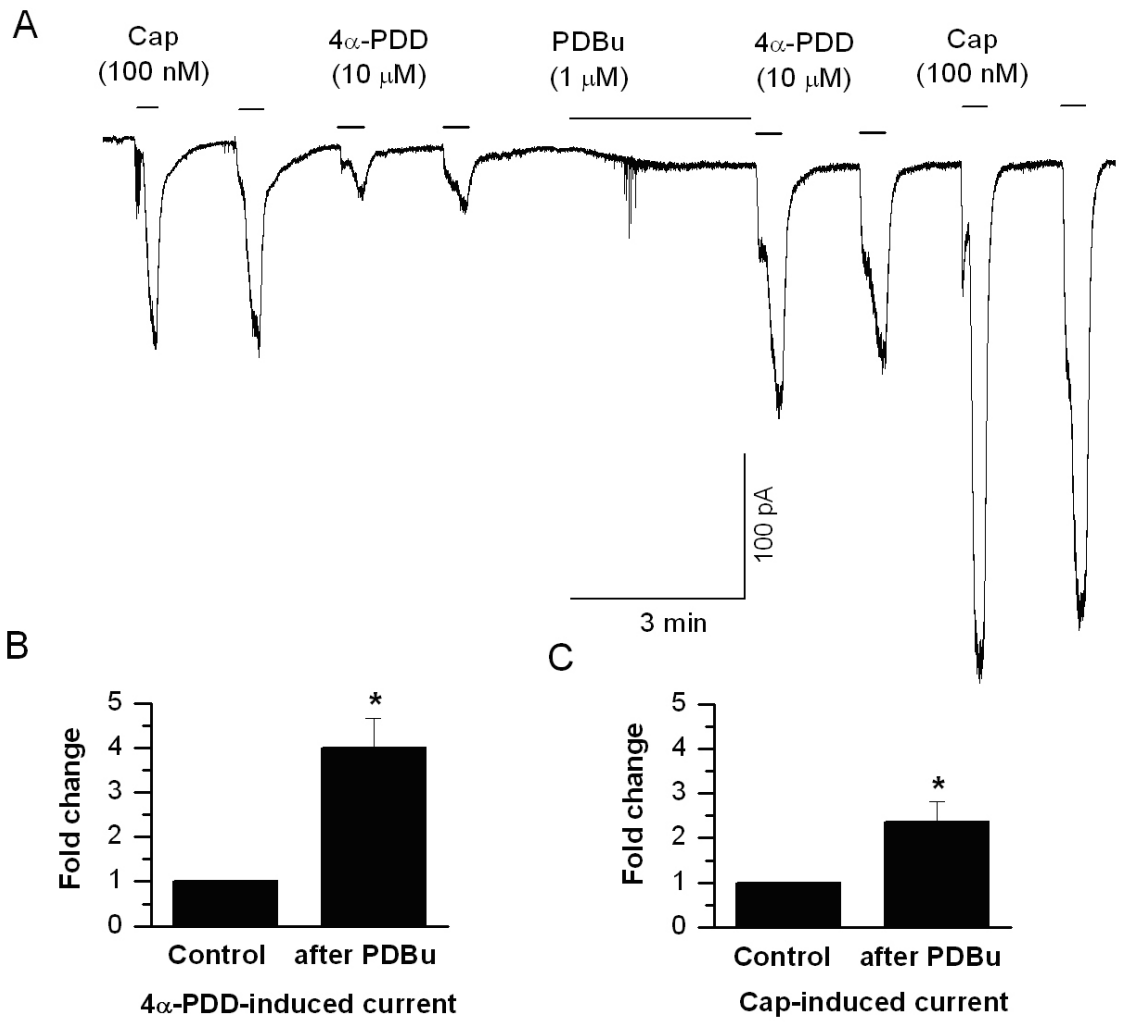
**Sensitization of TRPV4 and TRPV1 by PKC in cultured DRG neurons**. **A**. Both capsaicin (100 nM)-induced and 4α-PDD (10 μM)-induced currents are potentiated by a PKC activator, PDBu (1 μM). **B**, **C**. Summary graphs showing potentiation of 4α-PDD- and capsaicin-induced current activity by PDBu (1 μM; *n *= 11). The asterisk (*) represents *P *< 0.05 as compared to control.

In whole-cell current recordings, 4α-PDD (10 μM)-induced current was robustly potentiated following incubation with PDBu (1 μM, for 2 min), a PKC activator (4.00 ± 0.65-fold, *n *= 11, *P *< 0.05, Fig. 2A, B). As a positive control, we determined the potentiation of TRPV1 currents by PKC. As expected, capsaicin-evoked TRPV1 currents were significantly potentiated by PDBu (2.37 ± 0.45-fold, *n *= 11, *P *< 0.05, Fig. 2A, C).

**Figure 3:**
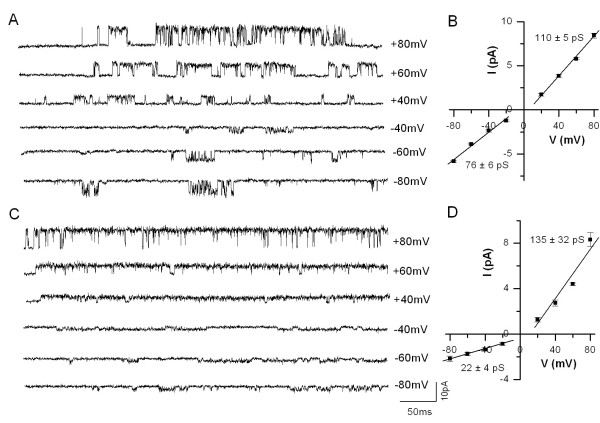
**Current-voltage relationship of 4α-PDD-evoked single-channel current in TRPV4 transfected HEK 293T cells and in DRG neurons natively expressing TRPV4**. **A**. Single-channel currents induced by 4α-PDD recorded at different membrane potentials from transfected HEK 293T cells. **B**. Current-voltage relationship shows that single-channel conductance is lower at negative potentials (76 ± 6 pS) as compared to positive potentials (110 ± 5 pS). **C**. Single-channel currents induced by 4α-PDD recorded at different membrane potentials from DRG neurons. **D**. Current-voltage relationship shows that the single-channel conductance is lower than that of cloned TRPV4 at negative potentials (22 ± 4 pS) as compared to positive potentials (135 ± 32 pS). The single-channel conductance contributes to the macroscopic current rectification.

Then, we recorded single-channel currents in cell-attached patches in DRG neurons and TRPV4 heterologously expressed in HEK 293T cells. Single-channel currents were recorded at positive and negative potentials. In DRG neurons at a holding potential of +60 mV, single-channel current had an amplitude of 4.40 ± 0.10 pA corresponding to a chord conductance of 73.3 ± 1.6 pS and at -60 mV single-channel current had an amplitude of 1.76 ± 0.15 pA corresponding to a chord conductance of 29.3 ± 2.5 pS (*n *= 3, Fig. 3). In similar experimental conditions, single-channel currents recorded at +60 mV from HEK cells had an amplitude of 5.70 ± 0.04 pA corresponding to a chord conductance of 95.0 ± 7.1 pS and at -60 mV had an amplitude of 3.81 ± 0.04 pA corresponding to a chord conductance of 63.5 ± 6.3 pS (*n *= 5, Fig. 3). It is clear from these experiments that TRPV4-mediated currents exhibited strong outward rectification. The outward rectification observed is similar to that observed with TRPV1 currents and we have described that outward rectification is due to both lower single-channel conductance and decreased open probability (P_o_) at negative membrane potentials [34]. In order to determine the mechanism of rectification, we have constructed current-voltage relationship by recording currents at different voltages form DRG neurons and HEK cells expressing TRPV4. The data points of outward and inward current limbs were fitted with linear functions to determine the slope conductance. The currents recorded from DRG neurons exhibited slope conductances of 135 ± 32 pS at positive potentials and 22 ± 4 pS at negative potentials (Fig. 3D). In similar experimental conditions, currents recorded from HEK cells expressing TRPV4 exhibited slope conductances of 110 ± 5 pS at positive potentials and 76 ± 6 pS at negative potentials (Fig. 3B). These results show that TRPV4 channels exhibit a strong outward rectification. The outward rectification exhibited by TRPV4 channels recorded from DRG neurons is more pronounced. The reason for this difference is not known, but it is possible that associated proteins in native TRPV4 channels [35] as reported in native TRPV1 channels [36] may contribute to rectification pattern.

In order to determine voltage dependency of the channel activity, we analyzed single-channel open probability at different membrane potentials from apparent single-channel patches. Single-channel patches were confirmed in patches that exhibited high P_o _by using the criteria of non-overlapping events when the P_o _was > 0.7. The P_o _at +60 mV and -60 mV is 0.84 ± 0.11 and 0.57 ± 0.11 (*n *= 3) in DRG neurons and 0.14 ± 0.04 and 0.07 ± 0.03 (*n *= 4) in HEK cells expressing TRPV4, respectively. These studies suggest that TRPV4 exhibits a milder voltage dependency in P_o _as compared to TRPV1.

**Figure 4:**
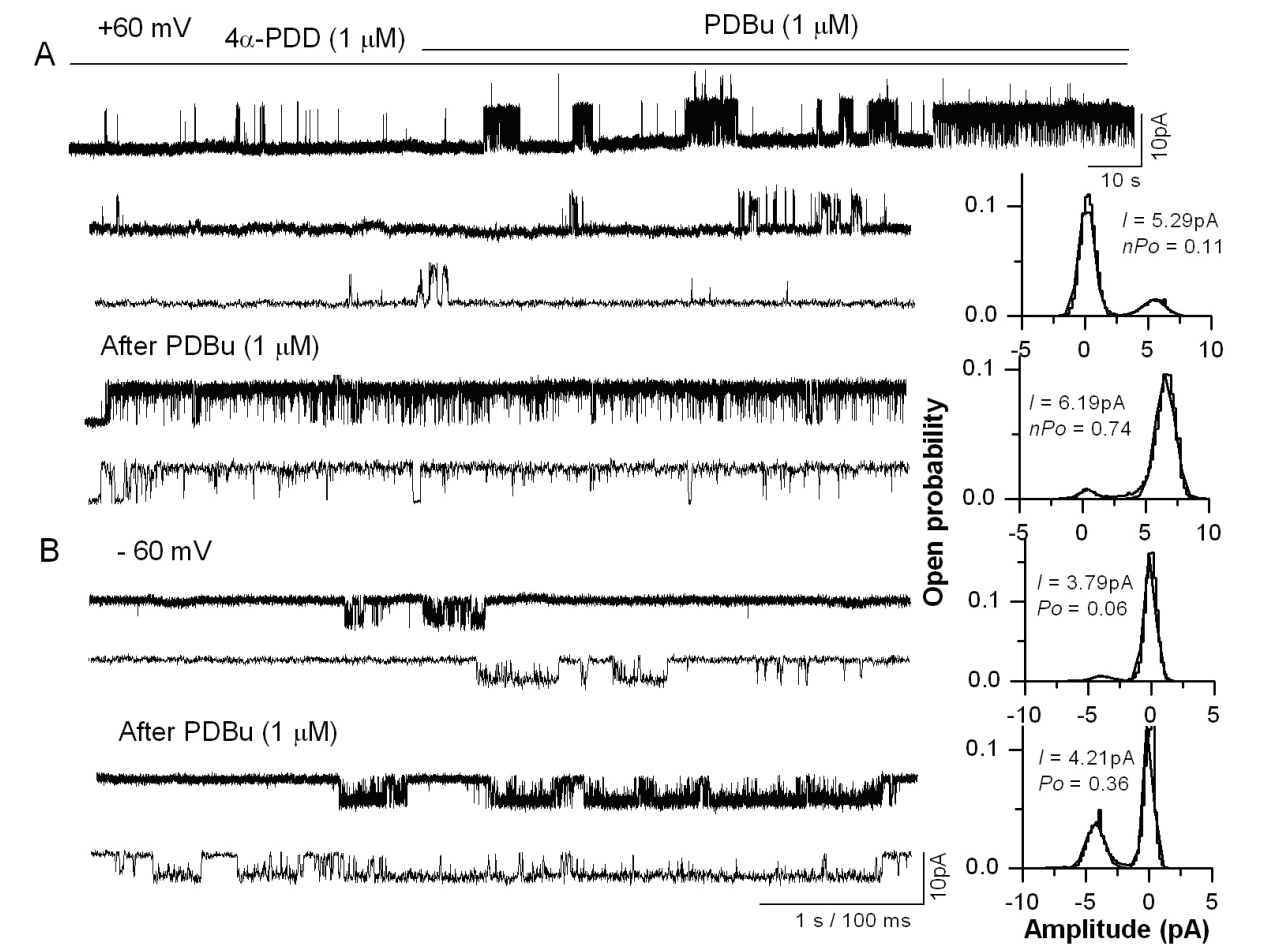
**Enhancement of TRPV4-mediated single-channel current activity by PKC**. **A**. Single-channel currents activated by 4α-PDD (1 μM) recorded at + 60 mV from a cell-attached patch on TRPV4 transfected HEK 293T cells before and after PDBu incubation. The traces are shown at higher time resolution below and the amplitude histograms are shown on the right. **B**. 4α-PDD-induced single-channel currents recorded at – 60 mV before and after PDBu incubation. The traces are shown at a higher time resolution below and the amplitude histograms are shown on the right.

In order to determine the role of PKC, cell-attached patches were formed with the pipette containing 4α-PDD (1 μM) and the cells were exposed to a PKC activator, PDBu (1 μM). In cell-attached patches from TRPV4 expressing HEK 293T cells, single-channel activity induced by 4α-PDD (1 μM) was significantly enhanced by the incubation of the cells with PDBu (1 μM). The open probability was significantly increased as compared to patches, in which the cells were not incubated with PDBu (control, 0.096 ± 0.023; PDBu, 0.507 ± 0.086; *n *= 7, *P *< 0.05, Fig. 4). In order to confirm the specificity of PKC action, the cells were incubated with a selective PKC inhibitor, BIM (500 nM). BIM significantly reduced the increase in the P_o _induced by PDBu (PDBu, 0.476 ± 0.113; BIM, 0.074 ± 0.035, *n *= 5, *P *< 0.05). These results indicate that TRPV4 can be sensitized by activation of PKC.

### Role of TRPV4 expressed in the central terminals of the spinal cord

**Figure 5:**
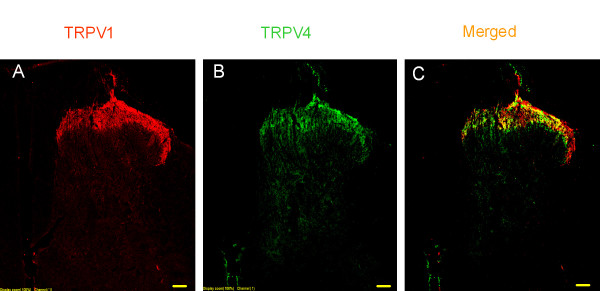
**TRPV1 and TRPV4 are expressed at the central terminals and are co-localized in spinal dorsal horn**. **A**. Immunostaining of TRPV1 in dorsal horn. **B**. Immunostaining of TRPV4 in dorsal horn. **C**. Merged image showing the extent of co-localization in yellow. Bar = 300 μm.

**Figure 6:**
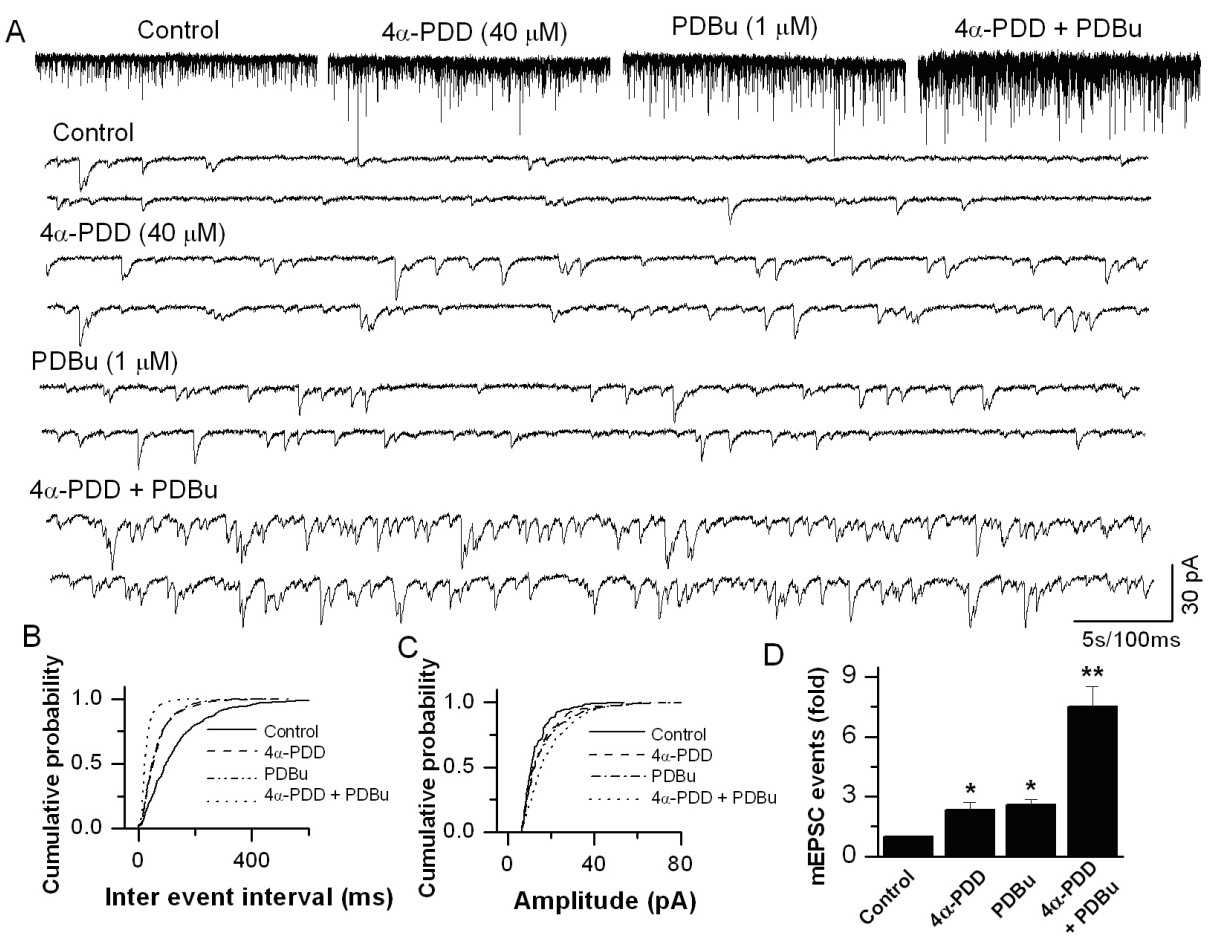
**Potentiation of 4α-PDD-induced changes in synaptic transmission by PDBu in DRG-DH co-cultures**. **A**. Application of 4α-PDD (40 μM) increases the frequency of mEPSCs and this action is enhanced by incubation with PDBu (1 μM). Synaptic currents are shown at a higher time resolution below. **B**. Cumulative probability plot indicates an increase in the frequency of synaptic events with 4α-PDD, which is further significantly enhanced following by PDBu incubation. **C**. The increase in frequency of events is not accompanied by a change in the amplitude. **D**. Summary graphs showing 4α-PDD-induced an increase in mEPSC frequency (*n *= 5) and potentiation by PDBu (*n *= 3). The asterisk (*) represents *P *< 0.05 as compared to control; (**) represents *P *< 0.05 as compared to 4α-PDD group.

Immunohistochemical data showed that TRPV1 and TRPV4 are expressed not only in the DRG neuronal cell bodies, but also expressed in central sensory nerve terminals at laminae I and II of the spinal dorsal horn (Fig. 5). In order to determine the role of TRPV4 expressed in the spinal cord, we studied the modulation of synaptic transmission by TRPV4 activation in DRG-DH neurons grown in culture. In DRG-DH co-cultures, synaptic connections are formed between DRG and DH neurons and synaptic currents can be recorded by voltage clamping the DH neurons. In a previous study from our lab has shown that TRPV1 activation increases mEPSC frequency without affecting the amplitude at the first sensory synapse [25]. We have also reported that only DRG-DH co-cultures responded to capsaicin application by increasing the frequency of mEPSCs. In similar experimental conditions, application of capsaicin to DH-only cultures did not induce a response suggesting the expression of TRPV1 only at the sensory nerve terminals. Application of 4α-PDD (40 μM) increased the frequency of mEPSCs significantly (2.33 ± 0.39-fold, *n *= 5, *P *< 0.05, Fig. 6), while there was no change in the amplitude of mEPSCs, suggesting that TRPV4 activation causes enhanced glutamate release from the presynaptic terminals. To determine whether TRPV4-mediated increase in mEPSCs is modulated by PKC, the neurons were incubated with PDBu (1 μM). A confounding issue is that PDBu by itself has been shown to increase the mEPSC frequency by binding to a site, independent of phosphorylation [25,37-40]; therefore, first we quantitated PDBu-mediated increase in the frequency of mEPSCs. PDBu alone significantly increased the frequency (2.58 ± 0.25-fold, *n *= 3, *P *< 0.05, Fig. 6). However, application of 4α-PDD + PDBu synergistically increased the frequency of TRPV4-induced mEPSCs (7.51 ± 0.99-fold, *n *= 3, *P *< 0.05, Fig. 6). These results suggest that TRPV4 activation modulates synaptic transmission, which is further enhanced by activation of PKC.

### Modulation of synaptic transmission by 4α-PDD in hippocampal neurons

**Figure 7:**
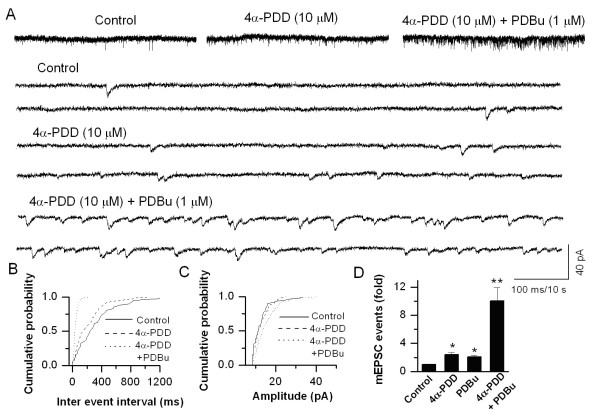
**Enhancement of 4α-PDD-induced increase in synaptic transmission by PDBu in cultured hippocampal neurons**. **A**. Application of 4α-PDD (10 μM) increases the frequency of mEPSCs, which is further potentiated by PDBu. The synaptic events are shown at a higher time resolution below. **B**. Cumulative probability plot shows a decrease in inter-event interval. **C**. The increase in frequency is not accompanied by a change in the amplitude. **D**. Summary graph showing 4α-PDD induces an increase in mEPSC frequency and this action is enhanced by PDBu (*n *= 7). The asterisk (*) represents *P *< 0.05 as compared to control; (**) represents *P *< 0.05 as compared to 4α-PDD group.

TRP channels are also expressed in certain regions of the CNS. TRPV1 and TRPV4 expression has been shown in the hippocampus [29,41-43]. Rat embryonic cultured hippocampal neurons were used to study the modulation of synaptic transmission by activation of TRPV4. We have selectively voltage-clamped hippocampal pyramidal neurons identified by their morphology. Using whole-cell recordings, application of 4α-PDD (10 μM) induced an increase in the frequency of mEPSCs, which was not accompanied by a change in the amplitude. The frequency of mEPSCs was increased 2.40 ± 0.26-fold (*n *= 7, *P *< 0.05) following application of 4α-PDD (Fig. 7). As observed in DRG-DH cultures, application of 4α-PDD + PDBu synergistically potentiated TRPV4-mediated response (10.06 ± 1.91-fold, *n *= 7, *P *< 0.05, Fig. 7). These experiments suggest that TRPV4 activation exerts an effect presynaptically and modulates synaptic transmission. Intriguingly, application of capsaicin (1 μM) in voltage-clamped cultured hippocampal pyramidal neurons neither changed the frequency (1.11 ± 0.10-fold, *n *= 6) nor the amplitude of mEPSCs. Although TRPV1 expression has been shown in the hippocampus [41,42], the function has not been studied extensively. Recently, a form of long term depression (LTD) has been attributed to activation of TRPV1 in the hippocampus [29].

## Discussion

TRPV1 has been extensively studied and has been shown to be involved in inflammatory thermal hypersensitivity. In this study, we have shown that: 1) TRPV1 and TRPV4 are co-expressed in a population of DRG neurons and their terminals in spinal dorsal horn; 2) TRPV4 can be sensitized by activation of PKC in DRG neurons similar to that of TRPV1; 3) Activation of TRPV4 modulates synaptic transmission at the first sensory synapse in the spinal cord, which is enhanced by activation of PKC similar to that of TRPV1; 4) TRPV4, but not TRPV1 activation modulates synaptic transmission between hippocampal neurons.

TRPV4 has been demonstrated to be activated by heat (> 27°C) and TRPV4-mediated Ca^2+ ^influx is strongly enhanced at 37°C in a PKC-dependent and -independent manner [2,33]. PKC activation by phorbol ester derivatives induced Ca^2+ ^influx in HEK 293 cells transfected with human TRPV4 cDNA and exposure to a hypotonic solution after phorbol myristate acetate incubation further increased intracellular Ca^2+ ^[32]. Furthermore, Alessandri-Haber et al. reported that hypotonicity-induced Ca^2+ ^influx was reduced by a PKCε inhibitor in DRG neurons [21].

We have conducted experiments at room temperature to avoid temperature effects and have studied whether 4α-PDD-induced TRPV4 responses could be modulated by activation of PKC. We have found that 4α-PDD-induced TRPV4 channel activity is further potentiated by PKC in whole-cell and single-channel recordings from DRG neurons and HEK cells expressing TRPV4. The results suggest that phosphorylation of TRPV4 by PKC is capable of sensitizing this channel. Single-channel recordings show that the potentiation is due to an increase in the open probability. Our study shows that a population of DRG neurons co-expresses both TRPV1 and TRPV4. TRPV1 has been implicated to contribute to inflammatory thermal and chemical pain [44,45]. Given the finding that TRPV4 contributes to mechanical hyperalgesia in behavioral tests and TRPV1 is involved in inflammatory thermal hyperalgesia, expression of both of these channels in one neuron will synergistically modulate nociception.

A hall-mark characteristic of many TRP channels is outward rectification. We have shown that the outward rectification observed with TRPV1 channels is due to both reduced single-channel conductance and open probability at negative potentials [34]. Single-channel TRPV4 currents recorded from DRG neurons showed more pronounced outward rectification as compared to currents recorded from HEK cells. This may be due to associated channel protein such as PACSIN3 [35].

The synaptic transmission between DRG neurons and spinal DH neurons play a key role in pain processing. Glutamate is released from presynaptic terminal (the central terminal of DRG neurons) upon a variety of stimuli and binds to its postsynaptic receptors (NMDA and AMPA receptors). Any process that increases glutamate release (presynaptic effect) or augments AMPA and NMDA receptor function (postsynaptic effect) may underlie central sensitization. Application of 4α-PDD significantly increased the frequency of mEPSCs without affecting the amplitude suggests that synaptic transmission is modulated by a presynaptic locus of action. This is expected because in the spinal cord TRPV4 is expressed only at the central sensory nerve terminals. However, one could also envision a postsynaptic effect by the release of neuropeptides such as CGRP, SP and bradykinin during intense synaptic activity. It has been shown that activation of PKC by PDBu or diacylglycerol (DAG) enhances excitatory synaptic transmission in the hippocampus [37-40]. Munc 13-1 is an essential priming factor in synaptic vesicles and it has a DAG/PDBu binding C1 domain [40,46]. Munc 18-1 has been shown to be essential for presynaptic vesicle release and has been identified as a PKC substrate [47]. Activation of Munc 13-1 or Munc 18-1 results in synaptic vesicle release. These studies suggest that PDBu-induced potentiation of synaptic transmission can be both PKC-dependent and PKC-independent mechanisms [48]. Although PDBu can modulate synaptic transmission by itself, we observed that activation of TRPV4 and PKC, synergistically increased the frequency of mEPSCs as shown with TRPV1 [25]. Therefore, enhanced expression and function of TRPV4 will result in increased excitability of spinal dorsal horn neurons, which may contribute to central sensitization. In disease conditions such as diabetes, the expression and function of TRPV1 is altered in spinal dorsal horn [49]. Similarly, TRPV4 expression and function could be altered in disease conditions. The endogenous ligand for TRPV4 may be EETs, generated from AA by cytochrome P450 epoxygenase activation during cell swelling [20]. It has been shown that intrathecal administration of TRPV4 antisense oligonucleotides reduces mechanical hyperalgesia [15]. However, it is not clear the locus of action of the antisense oligonucleotides. Furthermore, centrally acting TRPV1 antagonist has been shown to be more effective [50], raising the possibility that antagonizing the central TRPs contributes to the overall analgesic action. Therefore, targeting TRPV4 at the central terminals may be a useful strategy to combat certain modalities of pain.

TRP channels are expressed in different regions of the brain. Both TRPV1 and TRPV4 are expressed in the hippocampus [41,43,51]. TRPV1 activation has been reported to be involved in augmentation of synaptic transmission in the hypothalamus, locus coeruleus, nucleus tractus solitarius, substantia nigra and spinal cord by the observation that the mEPSC frequency was enhanced following application of capsaicin [22,23,25-28,52,53]. Furthermore, in hippocampal interneurons, TRPV1 activation has been shown to mediate a form of LTD involving interneurons, which is absent in TPRV1 knockout mice. LTD was induced by the TRPV1 agonists and inhibited by TRPV1 antagonists [29]. TRPV1 knockout mice show reduced anxiety-related behavior and deficits in developing long-term potentiation [54]. In this study, we show that activation of TRPV4, but not TRPV1, modulates synaptic transmission in voltage-clamped cultured pyramidal hippocampal neurons, suggesting that TRPV4 might play a role in CNS function. TRPV1-mediated membrane currents in hippocampal neurons have not been characterized fully. Therefore, lack of TRPV1-mediated response in our experiments is possibly due to rapid desensitization of TRPV1, selective expression in specific neurons or ontogenic expression (embryonic vs. adult). Future experiments conducted on specific cell types and from embryonic vs. adult neurons may resolve this discrepancy.

## Conclusion

This study indicates that TRPV1 and TRPV4 are co-expressed in certain DRG neurons and TRPV4 can be sensitized by PKC not only in DRG neuronal cell bodies, but also in the central sensory and non-sensory nerve terminals. Co-expression of TRPV1 and TRPV4 ion channels, their modulation of synaptic transmission and their sensitization by PKC may synergistically play a role in nociception.

## Competing interests

The authors declare that they have no competing interests.

## Authors' contributions

DSC carried out the electrophysiological experiments, performed data analysis and prepared the manuscript. SQY carried out the immunohistochemical experiments. LSP designed the study and prepared the final manuscript. All the authors have read and approved the final manuscript.
